# Medical comorbidities and other factors associated with migraine among individuals with diabetes mellitus in Hungary: a cross-sectional study using European Health Interview Surveys 2009–2019

**DOI:** 10.3389/fendo.2024.1379127

**Published:** 2024-08-23

**Authors:** Eszter Varga, Amr Sayed Ghanem, Eszter Faludi, Chau Minh Nguyen, Nóra Kovács, Attila Csaba Nagy

**Affiliations:** ^1^ Department of Neurology, Faculty of Medicine, University of Debrecen, Debrecen, Hungary; ^2^ Department of Health Informatics, Faculty of Health Sciences, University of Debrecen, Debrecen, Hungary; ^3^ Department of Public Health and Epidemiology, Faculty of Medicine, University of Debrecen, Debrecen, Hungary

**Keywords:** headache disorders, disability, migraine, diabetes mellitus, European Health Interview Survey

## Abstract

**Introduction:**

Migraine, a debilitating neurological disorder characterized by recurrent headaches, affects over 1.1 billion individuals globally. Diabetes mellitus (DM), a chronic metabolic condition marked by high blood sugar levels, affects 463 million individuals according to the International Diabetes Federation. Our study aimed to evaluate the association between migraine and DM and to identify several demographic, socioeconomic, and lifestyle factors, as well as medical and psychiatric comorbidities, associated with migraine among individuals with DM.

**Methods:**

This cross-sectional study is based on data from the European Health Interview Surveys conducted in 2009, 2014, and 2019 in Hungary. Pearson’s chi-squared tests and multiple logistic regression models were used to assess associations. Statistical significance was set at p<0.05.

**Results:**

In multiple regression analyses, we found no significant association between DM and migraine after adjusting for socioeconomic status, various health conditions, and lifestyle factors (OR=0.84, 95% CI: 0.66-1.06). However, adults with DM who had comorbid conditions including stroke (OR=2.08, 95% CI: 1.06-4.08), low back pain (OR=3.52, 95% CI: 2.13-5.84), and depression (OR=4.91, 95% CI: 2.84-8.47) were significantly more likely to suffer from migraine.

**Discussion:**

Our study found no significant difference in the prevalence of migraine among adults with and without diabetes mellitus. However, several comorbidities were found to be significantly associated with migraine occurrence in those with DM. Thus, the study’s results highlight the need for proper management of diabetes, especially in terms of comorbidities, to mitigate migraine risk factors and improve patient outcomes.

## 1 Introduction

Migraine is characterized by recurrent attacks of pulsatile, moderate-severe intensity headache, located on one side of the head, and accompanied by photo- and phonophobia, and/or nausea/vomiting (1–3). The global prevalence of migraine is 14-15%, and recent studies have indicated an increasing temporal trend in the last decades (4, 5).

Migraine-related disability burden is substantial. Migraine accounts for 4.9% of total years lived with disability (YLDs). Migraine is the leading cause of disability among young adult women, and most frequently affects individuals during the most productive years. Migraine is associated with impaired quality of life (5–8).

Moreover, there is evidence that migraine is comorbid with a range of conditions including depression, anxiety, and/or pain disorders. Migraine is also associated with increased prevalence of cardiovascular risk factors, such as hypertension, increased cholesterol level, smoking, and obesity (7, 9–13).

However, previous studies have yielded conflicting results regarding the association between migraine and diabetes mellitus (DM). Some studies have reported a lower (14, 15) prevalence of migraine in individuals with DM, while others have suggested similar (16–19) or higher (9, 20) rates compared with those without DM. Despite the limited and controversial results, some studies (19, 21) have suggested that socioeconomic factors and lifestyle habits may play a role in the link between migraine and DM. The possible underlying mechanisms involved in this potential link are not completely understood and require additional investigation (21).

Due to the increasing burden of migraine and DM worldwide, a better understanding of the complex relationship between these conditions, as well as examining potential risk factors could help inform clinical care. Our study aimed to estimate the prevalence of migraine in individuals with DM in a representative sample of the Hungarian adult population and to evaluate the association between migraine and several demographic, socioeconomic, and lifestyle factors, as well as medical and psychiatric comorbidities, among individuals with DM.

## 2 Materials and methods

### 2.1 Study design and study population

A cross-sectional study was carried out using data from the European Health Interview Surveys (EHIS) conducted in Hungary in 2009, 2014, and 2019. The datasets were obtained from the Hungarian Central Statistical Office (22). Individualized weights were applied to address non-response bias and achieve a representative sample to the Hungarian population (23). A standardized questionnaire, used across all participating EU Member States, was utilized to collect comprehensive data on health status. We integrated data from all three survey years including difference participants from each wave to perform the analysis to increase the statistical power of the results.

The final dataset consists of 12,025 participants living in private households. Individuals aged 35 years and older were included in this study.

The study was conducted with strict adherence to ethical guidelines, following the principles set forth in the Declaration of Helsinki. Ethical approval was secured from the Ethics Committee of the University of Debrecen, under the approval number 5609-2020, ensuring compliance with Regulation 2016/679.

### 2.2 Variables

Self-reported information on variables was used in the study. Participants were identified as subjects with diabetes who answered yes to both the following questions: “Have you had this condition (diabetes) in the past 12 months?” and “Has your physician confirmed the diagnosis?”. Respondents were considered as suffering from migraine if they answered yes to the following questions: “Have you had this condition (migraine) in the past 12 months?” and “Has your physician confirmed the diagnosis?”.

In the current study, a range of demographic, socio-economic, and health-related variables were analyzed. We examined socio-demographic factors, including gender (male, female) and age groups (35-64 years, and 65+ years). Educational background was categorized as completion of primary, secondary, and tertiary education. For income status, we analyzed the data across five groups: low income, lower middle-class income, middle-class income, upper middle-class income, and high income.

Self-perceived health status was measured on a scale from 1 to 3, where higher numbers indicated worse health. Self-reported height and weight were used to estimate the body mass index (BMI), which was categorized into three groups: underweight or normal (BMI < 25), overweight (25 ≤ BMI < 30), and obese (BMI ≥ 30).

Among lifestyle factors, smoking habits (smokers and non-smokers) and alcohol consumption (heavy drinkers, regular drinkers, occasional drinkers, and non-drinkers) were investigated.

Data on the presence of chronic conditions affecting the participants during the last 12 months were also collected regarding respiratory diseases (including asthma and bronchitis), acute myocardial infarction (AMI), coronary artery disease (CAD), stroke, high blood pressure, high cholesterol level, mental health disorders (including depression and anxiety), and low back pain.

### 2.3 Statistical analysis

Descriptive statistics were presented to describe the characteristics of the study populations with and without DM using absolute numbers and weighted proportions. Pearson’s chi-squared tests were used to test differences between groups. Multiple logistic regression models with binary outcomes were performed to measure the association between presence of DM and migraine, and to explore the socioeconomic, health-related and lifestyle factors, as well as medical and psychiatric comorbidities, associated with migraine among individuals with DM. Results of the logistic regression models are presented as odds ratios (OR) and 95% confidence intervals (CI) are presented. Sampling weights were applied using the “svy” command in Stata.

The statistical analyses were performed using STATA IC Version 18.0 (StataCorp. 2023. Stata Statistical Software: Release 18. College Station, TX: StataCorp LLC). A p-value less than 0.05 was considered statistically significant.

## 3 Results

More than half of individuals with DM were female (54.97%), and most of them belonged to the older age group (51.69%). A significantly better self-reported health status was reported by those with DM compared to those without DM (good rated health DM 36.74% vs non-DM 13.53%). Individuals with DM reported significantly higher BMI (obesity: 46.66% vs 24.38%, p<0.001), higher prevalence of comorbidities, lower education and income levels, and were more likely to be non-smokers (81.03% vs 73%, p<0.001) and non-drinkers (42.76% vs. 32.1%, p<0.001) than individuals without DM (**Table 1**).

**Table 1:** The distribution of health-related and sociodemographic factors among participants with and without diabetes mellitus.

		Total N=12,025	Without DM N=10,647	DM N=1,378	p-value
		N (%)*	N (%)*	N (%)*	
**Gender**	Male	5308 (45.31%)	4666 (45.03%)	642 (47.51%)	0.099
	Female	6717 (54.69%)	5981 (54.97%)	736 (52.49%)	
**Marital status**	Single	4651 (37.22%)	4093 (36.91%)	558 (39.71%)	0.054
	Married/in relationship	7266 (62.78%)	6460 (63.09%)	806 (60.29%)	
**Self-rated health**	Good	2006 (16.11%)	1512 (13.53%)	494 (36.74%)	**<0.001**
	Average	4491 (36.48%)	3833 (35.16%)	658 (47.03%)	
	Bad	5497 (47.41%)	5273 (51.3%)	224 (16.22%)	
**Comorbidities**	Asthma	707 (5.77%)	576 (5.32%)	131 (9.41%)	**<0.001**
	Bronchitis	684 (5.65%)	557 (5.19%)	127 (9.35%)	**<0.001**
	AMI	500 (4.05%)	367 (3.33%)	133 (9.78%)	**<0.001**
	CAD	912 (7.26%)	664 (5.96%)	248 (17.69%)	**<0.001**
	High blood pressure	5271 (42.32%)	4194 (37.9%)	1077 (77.79%)	**<0.001**
	Stroke	400 (3.24%)	304 (2.74%)	96 (7.23%)	**<0.001**
	Low back pain	3450 (28.11%)	2893 (26.58%)	557 (40.39%)	**<0.001**
	Depression	624 (5.09%)	506 (4.66%)	118 (8.52%)	**<0.001**
	High cholesterol level	1967 (16.03%)	1423 (13.14%)	544 (39.25%)	**<0.001**
	Migraine	886 (7.42%)	742 (7.06%)	144 (10.32%)	**<0.001**
**Age groups**	35-64 years	8135 (70.06%)	7497 (72.77%)	638 (48.31%)	**<0.001**
	65 years or older	3890 (29.94%)	3150 (27.23%)	740 (51.69%)	
**BMI**	Normal	3958 (33.59%)	3749 (35.8%)	209 (15.88%)	**<0.001**
	Overweight	4722 (39.56%)	4210 (39.82%)	512 (37.46%)	
	Obese	3267 (26.84%)	2622 (24.38%)	645 (46.66%)	
**Smoking status**	Non-smoker	8804 (73.89%)	7694 (73%)	1110 (81.03%)	**<0.001**
	Smoker	3056 (26.11%)	2808 (27.00%)	248 (18.97%)	
**Alcohol consumption**	Non-drinker	4075 (33.28%)	3486 (32.1%)	589 (42.76%)	**<0.001**
	Occasional drinker	4869 (41.38%)	4389 (42.15%)	480 (35.21%)	
	Regular drinker	2306 (19.72%)	2073 (20.05%)	233 (17.01%)	
	Heavy drinker	663 (5.622%)	597 (5.696%)	66 (5.025%)	
**Level of education**	Primary	5057 (41.6%)	4346 (40.25%)	711 (52.43%)	**<0.001**
	Secondary	4624 (37.91%)	4151 (38.47%)	473 (33.46%)	
	Tertiary	2339 (20.48%)	2146 (21.28%)	193 (14.11%)	
**Income quintiles**	1st quintile	2397 (19.63%)	2078 (19.11%)	319 (23.77%)	**<0.001**
	2nd quintile	2473 (19.98%)	2163 (19.82%)	310 (21.27%)	
	3rd quintile	2517 (20.85%)	2235 (20.89%)	282 (20.50%)	
	4th quintile	2529 (20.87%)	2248 (20.93%)	281 (20.43%)	
	5th quintile	2109 (18.66%)	1923 (19.24%)	186 (14.03%)	

*weighted proportions and unweighted numbers. Bold values indicate statistical significance (p < 0.05) based on Pearson’s chi-squared test.

Individuals with DM showed a significantly higher overall prevalence of migraine compared to those without DM (10.32% vs. 7.06%, p<0.001). Female participants reported a higher prevalence of migraine than males, particularly among those with DM (14.31% vs. 5.93%). Single individuals with DM reported a higher rate of migraine compared to those in a relationship (13.15% vs. 8.24%, p=0.023). Individuals with DM and depression had an increased prevalence of migraine (43.7%) than those without DM (32.81%) (p<0.001). In those with both DM and comorbidities including bronchitis, AMI, CAD, low back pain, and high cholesterol level, there was a significantly increased prevalence of migraine compared to those without DM. Regarding socioeconomic and lifestyle factors, a significantly higher migraine prevalence was reported among those with primary education (14.93% and 10.65% in DM and non-DM patients, p=0.012) compared with those with higher education, and among smokers (13.51% in DM and 7.6% in non-DM patients, p=0.006) compared with non-smokers (**Table 2**).

**Table 2:** Prevalence of migraine in various strata of participants with DM vs. without DM.

	Total	Total	Without DM	DM	p-value
		886 (7.42%)	742 (7.06%)	144 (10.32%)	<0.001
**Gender**	Male	5308 (4.31%)	4666 (4.08%)	642 (5.93%)	0.764
	Female	6717 (10.02%)	5981 (9.49%)	736 (14.31%)	
**Marital status**	Single	4651 (8.42%)	4093 (7.77%)	558 (13.15%)	**0.023**
	In a relationship	7266 (6.84%)	6460 (6.66%)	806 (8.24%)	
**Self-rated health**	Good	2006 (21.23%)	1512 (21.77%)	494 (19.58%)	**<0.001**
	Average	4491 (7.3%)	3833 (7.53%)	658 (5.92%)	
	Bad	5497 (2.85%)	5273 (2.88%)	224 (2.28%)	
**Comorbidities**	Asthma	707 (18.67%)	576 (18.07%)	131 (21.3%)	0.082
	Bronchitis	684 (21.29%)	557 (20.19%)	127 (26.14%)	**0.012**
	AMI	500 (20.29%)	367 (21.16%)	133 (17.89%)	**<0.001**
	CAD	912 (21.61%)	664 (22.22%)	248 (19.99%)	**<0.001**
	High blood pressure	5271 (10.08%)	4194 (9.75%)	1077 (11.38%)	0.096
	Stroke	400 (19.25%)	304 (18.32%)	96 (22.18%)	0.209
	Low back pain	3450 (15.94%)	2893 (15.3%)	557 (19.25%)	**<0.001**
	Depression	624 (34.87%)	506 (32.81%)	118 (43.7%)	**<0.001**
	High cholesterol level	1967 (13.78%)	1423 (14.02%)	544 (13.13%)	**0.004**
**Age groups**	35-64 years	8135 (7.08%)	7497 (6.72%)	638 (11.37%)	0.205
	65 years or older	3890 (8.23%)	3150 (7.97%)	740 (9.35%)	
**BMI**	Normal	3958 (7.8%)	3749 (7.57%)	209 (11.78%)	0.097
	Overweight	4722 (7.17%)	4210 (6.96%)	512 (8.92%)	
	Obese	3267 (7.39%)	2622 (6.52%)	645 (10.89%)	
**Smoking status**	Non-smoker	8804 (7.16%)	7694 (6.82%)	1110 (9.66%)	**0.006**
	Smoker	3056 (8.07%)	2808 (7.60%)	248 (13.51%)	
**Alcohol consumption**	Non-drinker	4075 (11.68%)	3486 (11.32%)	589 (13.85%)	0.356
	Occasional drinker	4869 (6.15%)	4389 (5.79%)	480 (9.49%)	
	Regular drinker	2306 (3.47%)	2073 (3.41%)	233 (3.95%)	
	Heavy drinker	663 (4.25%)	597 (3.79%)	66 (8.42%)	
**Level of education**	Primary	5057 (11.26%)	4346 (10.65%)	711 (14.93%)	**0.012**
	Secondary	4624 (5.0%)	4151 (4.96%)	473 (5.3%)	
	Tertiary	2339 (4.15%)	2146 (4.05%)	193 (5.2%)	
**Income**	1st quintile	2397 (19.63%)	2078 (19.11%)	319 (23.77%)	0.086
	2nd quintile	2473 (19.98%)	2163 (19.82%)	310 (21.27%)	
	3rd quintile	2517 (20.85%)	2235 (20.89%)	282 (20.50%)	
	4th quintile	2529 (20.87%)	2248 (20.93%)	281 (20.43%)	
	5th quintile	2109 (18.66%)	1923 (19.24%)	186 (14.03%)	

*weighted prevalences and unweighted numbers. Bold values indicate statistical significance (p<0.05) based on Pearson’s chi-squared test.

In a multiple regression analysis, adjusting for age and gender, we found a significant association between DM and migraine (OR=1.55, 95% CI: 1.26-1.90) in the total sample, suggesting that individuals with DM had a 55% higher likelihood of experiencing migraines compared to those without DM. However, when we further adjusted for a broader range of factors, including socioeconomic status, various health conditions, and lifestyle factors, this association was no longer significant (OR=0.84, 95% CI: 0.66-1.06). **Table 3** shows the results of multiple regression analysis among DM participants. Individuals with DM and with comorbid conditions including stroke (OR=2.08, 95% CI: 1.06-4.08), low back pain (OR=3.52, 95% CI: 2.13-5.84) and depression (OR=4.91, 95% CI: 2.84-8.47) were significantly more likely to suffer from migraine according to the results of a weighted multiple logistic regression model. Furthermore, in the DM population, individuals with secondary education reported significantly lower likelihood of having migraine (OR=0.43, 95% CI: 0.23-0.82) compared to those with primary education. The other variables showed no significant association with migraine prevalence (**Table 3**).

**Table 3:** Weighted multiple logistic regression models to identify the factors associated with migraine.

	OR (95%CI)
Gender		
Female/male	1.54 [0.88-2.72]
Marital status		
Married or in a relationship/single	0.80 [0.50-1.30]
Self-rated health		
Average/good	0.64 [0.4-1.02]
Bad/good	0.41 [0.15-1.15]
Comorbidities		
Asthma (yes/no)	1.00 [0.53-1.90]
Bronchitis (yes/no)	1.42 [0.70-2.89]
AMI (yes/no)	1.51 [0.86-2.65]
CAD (yes/no)	1.33 [0.83-2.15]
High blood pressure (yes/no)	1.07 [0.57-2.02]
Stroke (yes/no)	**2.08 [1.06-4.08]**
Low back pain (yes/no)	**3.52 [2.13-5.84]**
Depression (yes/no)	**4.91 [2.84-8.47]**
High cholesterol level (yes/no)	1.29 [0.84-1.99]
Age group		
65 years or older/35-64 years	0.98 [0.97-1.00]
BMI		
Overweight/normal	0.65 [0.35-1.21]
Obese/normal	0.65 [0.35-1.20]
Smoking		
Smoker/non-smoker	1.05 [0.79-1.41]
Alcohol consumption		
Occasionally/non-drinker	1.14 [0.68-1.89]
Regular drinker/non-drinker	0.56 [0.22-1.44]
Heavy drinker/non-drinker)	0.83 [0.22-3.17]
Education		
Secondary/primary	**0.43 [0.23-0.82]**
Tertiary/primary	0.50 [0.21-1.19]
Income		
2^nd^ quintile/1^st^ quintile	0.83 [0.46-1.49]
3^rd^ quintile/1^st^ quintile	0.76 [0.41-1.39]
4^th^ quintile/1^st^ quintile	0.67 [0.32-1.39]
5^th^ quintile/1^st^ quintile	1.08 [0.50-2.37]
Year		
2014/2009	0.71 [0.43-1.16]
2019/2009	0.81 [0.45-1.46]

OR, odds ratio; CI, confidence interval. Bold values indicate statistical significance (p<0.05).

## 4 Discussion

Though our study found an association between DM and migraine when adjusting for age and gender, our study found no significant differences in the prevalence of migraine among adults with DM compared to those without DM after adjusting for several socioeconomic, lifestyle, and health-related variables, which is consistent with the literature (19). We also showed that among those with DM, stroke, low back pain, depression and educational attainment were significantly associated with increased migraine prevalence.

In our study, we observed a higher prevalence of migraine among females with DM compared to those without DM. Migraine occurs more commonly in the female population, with a prevalence three times higher in females compared to males. Additionally, after menopause, the prevalence of migraine in females decreases (24). The observed gender and temporal disparity of migraine is related in part to the complex role of estrogen in migraine pathophysiology. Estrogen influences serotonin reuptake and regulation, and fluctuations of estrogen in the menstrual cycle can affect migraine frequency (25, 26). Therefore, stabilization of estrogen levels post-menopause is associated with decreased migraine frequency (26, 27, 29, 30).

Our study did not identify an increased risk of diabetes and migraine when adjusting for demographics and lifestyle factors. However, possible links between migraine and DM exist. Several studies have investigated the role of glucose dysregulation in the pathophysiology of migraine and headache disorders in DM. A recent study found that plasma glucose levels were higher in individuals with migraine (with and without aura) during migraine attacks (31). In addition, previous research has suggested that shared genetics and biological mechanisms may contribute to migraine and glucose-related traits (32, 33). Insulin plays a role in the pathophysiological mechanism in migraine, as it is associated with glucose metabolism and directly affects the secretion of gonadotropins by the hypothalamus. In addition, chronic sleep deprivation in those with migraine can cause hyperinsulinism due to higher postprandial glucose levels with abnormal insulin sensitivity (34). As a consequence, good sleep hygiene, as well as dietary patterns that include small, frequent meals high in protein and vegetables and low in carbohydrates may contribute to migraine prevention by minimizing blood glucose fluctuations (35).

The overlap of comorbid conditions with migraine in individuals with DM, including depression and chronic pain, is well-recognized in the scientific literature. Our study’s finding that migraine occurrence is more common in individuals with depression is consistent with a growing body of research underscoring the bidirectional relationship between migraine and depression (36). According to Holt et al. (2014), up to one third of individuals with DM experience depressive symptoms, leading to decreased quality of life (37), particularly affecting those of younger age, female gender, and lower educational levels.

Our study showed that in individuals with DM, lower back pain significantly increased the risk of comorbid migraine, which is consistent with the literature (38–40). This may be related in part to frequent use of acute analgesics for headache and other pain conditions, which can lead to medication overuse headache as a complicating factor for those with an underlying headache disorder (38, 39). Furthermore, experiencing one chronic pain condition has been associated with an increased risk of an additional chronic pain condition, raising the possibility of shared underlying mechanisms. Central sensitization to pain, in which the nervous system develops altered responsiveness to and processing of pain stimuli, leads to increased pain perception (40). For individuals with DM alone, Aldossari et al. (2020) highlighted a correlation with chronic pain, which may be related in part to comorbid diabetic neuropathy and inflammation (41).

The present study found that in those with DM and history of stroke, there is an increased risk of migraine compared to those without DM. The literature suggests that migraine with aura is associated with an increased risk of stroke (42) due to complex, multifactorial etiologies, related in part to alterations in cerebral blood flow, inflammation, platelet hyperaggregability, and endothelial activation (43). However, only 20% of individuals with migraine have migraine with aura (44). The increased prevalence of migraine in those suffering from both stroke and DM may be explained by the presence of metabolic risk factors including hypertension and obesity, which are known to be associated with migraine (45). Another explanation may be that individuals with DM and stroke may have reduced physical activity levels. Regular physical activity has a prophylactic effect on the frequency of migraine (46).

### 4.1 Strength and limitation

The strength of this study lies in its use of a representative database that covers a large and diverse population. Additionally, the availability of data on sociodemographic characteristics, lifestyle, and health-related variables allowed us to account for potential confounding factors in our analyses. Probability sampling and weighting contributed to a reduced non-response bias and external validity of the study.

However, the study has several limitations. First, the questionnaire was self-reported, so there may have been variable biases and inaccuracies in the data used. Second, the diagnoses of migraine and diabetes were based on self-reporting, relying on recall. Thus, bias from underreporting or overreporting may have occurred. Third, the cross-sectional study design does not allow causal inferences. In addition, regarding the diagnosis of stroke, the distinction between ischemic and hemorrhagic stroke was not made. Diabetes severity was also not noted, and type 1 and type 2 DM were not differentiated.

## 5 Conclusion

The study did not find an increased risk of diabetes and migraine when adjusting for demographics and lifestyle factors. However, there were increased risks of comorbidities of low back pain, stroke, and depression in individuals with DM and migraine compared to those without DM. This highlights the complexities of these conditions, and raises the importance of addressing vascular risk factors and chronic pain in those with DM and migraine. Further longitudinal and perhaps genetic research is needed to unravel the underlying mechanisms between DM and migraine.

## Data Availability

The datasets of the European Health Interview Survey for this study are available upon request from the Hungarian Central Statistical Office (https://www.ksh.hu). Requests to access these datasets should be directed to Karolyne Tokaji, Karolyne.Tokaji@ksh.hu.
